# Complications of Deep Venous Stenting and Their Management

**DOI:** 10.1007/s00270-024-03853-3

**Published:** 2024-11-04

**Authors:** Rachael I. Morris, Taha Khan, Stephen A. Black

**Affiliations:** Academic Department of Vascular Surgery, School of Cardiovascular and Metabolic Medicine and Sciences, King’s College London, St Thomas’ Hospital, 1st Floor North Wing, Westminster Bridge Road, London, SE1 7EH UK

**Keywords:** Deep vein thrombosis, Post-thrombotic syndrome, Venous stenting

## Abstract

Complications after iliofemoral and inferior vena cava stenting are rare, and most can be managed effectively without significant long-term consequences for the patient. Nevertheless, the procedure is not without risk, and clinicians considering starting a venous practice must be aware of the range of complications that can occur, which range from minor access site bleeds to fatal arrhythmia from stent migration to the heart. Most complications can be avoided with appropriate patient selection, stent sizing, and careful access and deployment techniques.

## Introduction

Iliocaval venous stenting has the potential to significantly improve clinical symptoms and quality of life for patients suffering from post-thrombotic syndrome [1–4]. The procedure has grown in popularity in recent years with the introduction of dedicated venous stents [5–8]. While long-term follow-up data for these devices are lacking, iliocaval stenting is generally thought to be a low-risk procedure, with IDE (investigational device exemption) trials reporting serious adverse event rates between 1.2% [6] and 6.5% [8]. These studies, however, typically include relatively small numbers of patients who are highly selected and treated in high-volume specialist centres. The true incidence of complications is likely higher when procedures take place in so-called ‘real world’ conditions. Although serious complications are rarely reported, there are situations, where these can become life-threatening, for instance with vessel rupture or stent migration to the heart [6, 9]. It is important, therefore, for all clinicians involved in treating patients undergoing these procedures to understand the potential complications, how to minimise the risk of these occurring and to be aware of appropriate management strategies.

## Access Site Complications

Access site complications including haematoma, pseudoaneurysm, iatrogenic arteriovenous (AV) fistula, lymphocele and infection may occur in up to 5% of patients [9–13]. In most cases, this is related to suboptimal access technique. For chronic iliofemoral disease, femoral vein access is preferable, although in some cases of severe disease or IVC involement, jugular venous access may be required. In acute cases, restoration of flow in more distal vessels can be needed and popliteal venous access is advised. Access should always be gained with ultrasound guidance. In our centre’s experience, using a micropuncture needle access kit, with sequential dilation of the tract starting with a 4F sheath and progressing to 6F and 9F as dictated by device requirements can help to minimise the potential risk associated with accidental arterial puncture. Compression is applied at the end of the procedure with a compression dressing over the access site, and with thigh-length compression stockings. When access is limited to 10F sheaths we have found that bleeding and haematoma are uncommon and closure devices are not needed.

Most access site haematomas will resolve with conservative management; however, haemoglobin should be monitored as transfusion or surgical evacuation for larger haematomas may be required in some cases. Anticoagulation will usually be continued to avoid the risk of in-stent-thrombosis, which is highest in the first 2-weeks postprocedure [14]. Iatrogenic AV fistulas usually resolve spontaneously and are managed conservatively, since the increased flow through the stent system from the fistula can be helpful for maintaining stent patency. The risk of seromas and access-site infections is higher in patients requiring hybrid surgical procedures involving open endophlebectomy [15, 16]. Severe inflow disease or other complicating factors such as previous failed interventions are usually present in patients being considered for endophlebectomy, and a thorough discussion with the patient regarding the higher risks of stent occlusion and groin wound complications is necessary before intervention. If a hybrid procedure is considered appropriate, high-level compression (class 2 or above) would be appropriate to reduce the risk of seromas and lymphoceles. Early drain usage and surgical haematoma evacuation can also be helpful in such cases. Infections may be managed with antibiotics (which initially may be broad spectrum but should be targeted to the specific organisms once wound culture results are available) vacuum assisted closure (VAC) dressings and surgical wash-out in some cases.

## Device-Related Complications

### Stent Migration

Stent migration is one of the most feared device-related complications of venous stenting. Literature on the rate of stent migration is sparse, however, one recent systematic review describes details of 54 cases [17], and a review of dedicated venous stents reported a migration rate of 0.17% [18]. Migration most commonly occurs in patients treated for non-thrombotic iliac vein lesions, and results from either under-sizing of the stent length or diameter, or from placing a stent in a patient who does not have a fixed lesion [17]. Migration can be within the iliac vein, to the inferior vena cava (IVC), or in the most serious cases, the right atrium, ventricle, or pulmonary artery, which can be life-threatening, and may require open surgical retrieval. Migration to the heart can result in cardiac arrest secondary to arrhythmia [17], perforation of the myocardium, transection of the distal right coronary artery and extensive damage to the tricuspid valve requiring major reconstructive surgery has also been described [19].

Migration may occur during the procedure, which can usually be managed immediately with endovascular techniques, or patients may present days, weeks, or months after the procedure with chest pain or arrhythmias. In some cases, stent migration does not cause symptoms and can be picked up as an incidental finding on routine surveillance imaging. If stent migration occurs during the procedure and is confined to the iliac veins or IVC, it can be managed by placement of an additional stent caudal to the site of migration to anchor it, or repositioning can be attempted using a snare or angioplasty balloon. Endovascular management may also be attempted in cases of delayed presentation but is less successful if the stent has already been embedded into the migrated position.

When appropriately sized stents are placed into true stenotic lesions, migration is rare [17]. As IVC and iliofemoral venous diameter can vary with hydration status and position [20, 21], it is important to assess the lesion when the patient is not dehydrated (ideally 1L of water in the hour before the scan, ensure the patient is aware that fasting is not required before imaging) and to use multiple imaging modalities with the patient in different positions to establish whether a true stenotic lesion exists. In some cases, there may be a positional compression that changes with respiration or ambulation. Duplex ultrasound is particularly useful as the patient can be scanned in the standing position and changes with respiration can be evaluated. Although invasive, intravascular ultrasound (IVUS) can be an invaluable diagnostic technique for dynamic evaluation of lesions, for instance, asking the patient to perform a valsalva manoeuvre, whiile IVUS is positioned at the vessel crossing may uncover changes in the degree of compression. Balloon pull-back, where the balloon is inflated beyond the lesion and gently pulled back can also indicate, whether there is a clinically relevant compression or stenosis (i.e. if the balloon pulls back easily then the need for stenting should be reassessed). Multiplanar venography can also demonstrate whether there are significant collaterals (suggesting a fixed lesion). A fixed stenosis of > 60% is recommended as an appropriate threshold for stenting [22].

When the presence of an obstructive lesion is established, stents should be sized to the same size as the native healthy vessel—if the contralateral side is healthy this can be used to assess the target vessel diameter, or if not approximately 14–16 mm for the common iliac vein, 12–14 mm for the external iliac vein and 10–12 mm for the common femoral vein [23]. As per the device instructions, the chosen stent should be 1–2 mm larger than the calculated vein diameter. Stents > 16 mm are rarely required, and if an 18 mm stent seems necessary this is an indication to consider the patient’s symptoms and imaging, and whether stenting is likely to bring improvement. Although longer stents result in a greater metal load and more coverage of the confluence with the internal iliac vein, to avoid migration, it is advised where possible to anchor the stent in the mid-segment of the external iliac vein to ensure that it follows the anatomical curvature through the pelvis.

### Stent Fracture

**Fig. 1:**
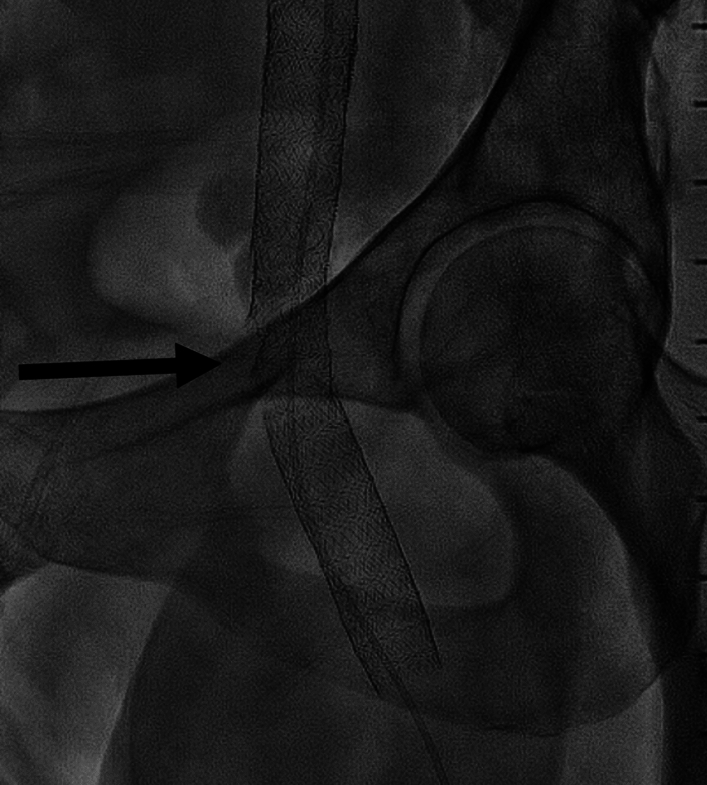
Iliac vein stent fracture of a closed-cell stent at the inguinal ligament. Arrow indicates the level of the stent fracture

Stent fractures can occur during, or at any time after the initial procedure. They occur more commonly in closed-cell stents extending into the common femoral vein [14], and the fracture site is usually adjacent to the femoral head, approximately 1 cm below the inguinal ligament (Fig. 1). The mechanism of stent fracture is not completely understood, but it may be related to compression from the inguinal ligament, or from the pubic rami with repeated flexion and extension movements [24]. The incidence of stent fracture reported ranges from 0% [5] to 3.3% [6] in the IDE trials. In some cases, stent fracture is asymptomatic and can be monitored without the need for intervention, but in severe (multiple breaks with deformation or complete separation) or symptomatic cases (recurrence of preoperative symptoms or new pain) further procedures may be required. Relining with an additional stent may be sufficient, however, in our centre’s experience, some patients have had repeated fractures requiring multiple re-interventions. In these refractory symptomatic cases division of the inguinal ligament has been attempted but does not always lead to improvement. Open excision of the stented segments can be undertaken if other management strategies are unsuccessful, and the patient’s symptoms are severe [25].

Although open-cell stents are less likely to fracture [14], the nature of the stent changes at areas of overlap, becoming more rigid at these points which theoretically increases the risk of fracture, therefore optimal placement of stents is essential and it is important to avoid overlapping stents at the inguinal ligament or adjacent to the femoral head [26].

### Stent Compression

**Fig. 2:**
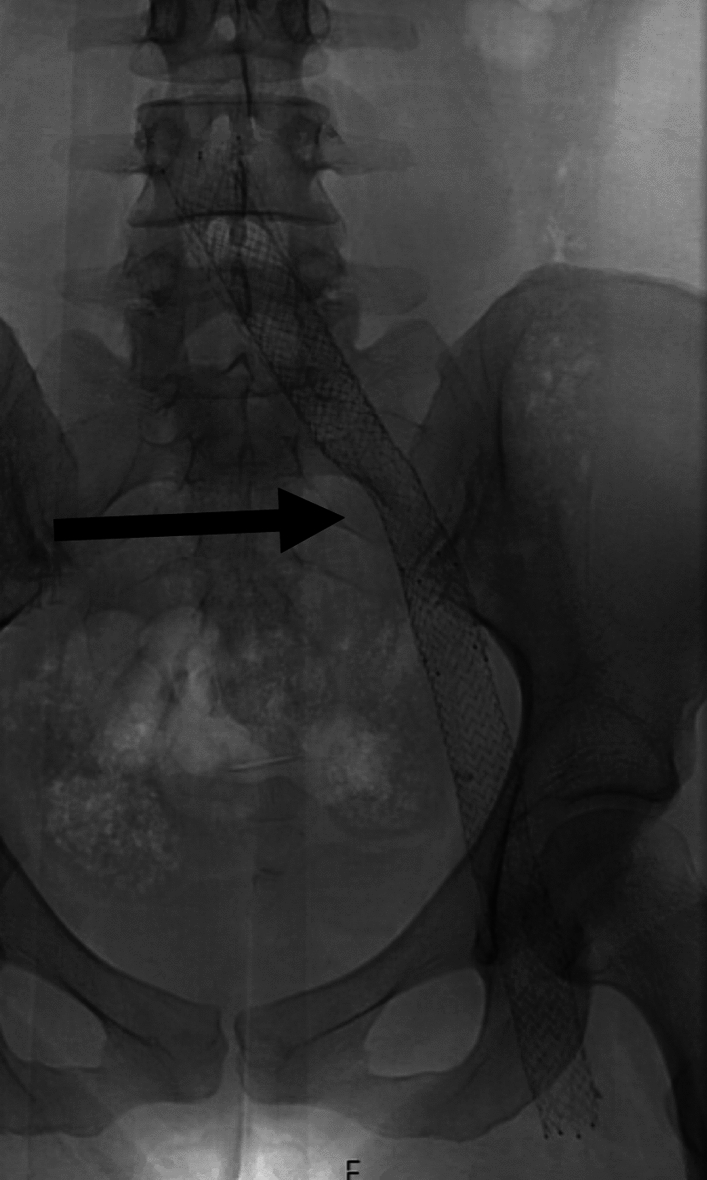
A patient with left common iliac vein stent compression of an open-cell stent from overlying left common iliac artery. Arrow indicates the area of compressed venous stent

Stent compression most commonly occurs in the left common iliac vein at the right common iliac artery crossing point but may also be seen on the right hand side, at the inguinal ligament, or, in patients undergoing suprarenal IVC stenting, the liver and diaphragm (Fig. 2). Compression may be noted during the procedure, or during follow-up, it is managed by relining with an additional stent.

### Fistulation to Other Organs

Fistulation between the target vein and surrounding organs is relatively rare but can carry high morbidity and mortality [27]. It results from adhesions of surrounding structures to the iliac vein or IVC, which are usually related to previous surgical interventions such as retroperitoneal dissection (e.g. malignancy) or radiotherapy. For example bowel may be adherent to the occluded venous tract and the stents can erode into these segments causing bowel-caval fistula. This can happen during ballooning of the vein or postdilation of the stent. Patients may develop a sudden septic response, and the diagnosis can be confirmed with cross-sectional imaging. Early detection and intervention are crucial, and immediate surgical repair should be considered if possible. In patients with a history of major pelvic or abdominal surgery it is important to identify the position of surrounding organs with cross-sectional imaging before intervention. IVUS can be useful intra-operatively to confirm if any organs are nearby. If fistulation is considered a risk, balloon dilation should be sequential, starting with a 6 mm balloon before proceeding to 10 mm and 14 mm.

## Other Device-Related Complications

Additional device-related complications that may occur during the procedure include balloon rupture and failure of stent deployment. The incidence of balloon rupture increases if the balloon is inflated repeatedly or beyond the manufacturer’s recommended number of cycles. If balloon rupture occurs, the tear is usually longitudinal, the risk of detachment is minimal and retrieval is straightforward, however, if circumferential rupture occurs detachment is more likely and fragments must be carefully retrieved to prevent embolization. In such cases, a snare may be used to capture the residual fragments.

Stent deployment failure, specifically failure of the stent to open completely upon deployment was noted to affect a number of the Bard Venovo stents, resulting in a temporary withdrawal from the market between 2021 and 2022 [28, 29]. This does not commonly result in serious complications for the patient [30] and is managed by the deployment of a new stent or the use of a balloon to complete stent expansion. Deployment issues have been sporadically reported but not published with other devices, and these appear largely related to technical aspects of deployment. The reader is encouraged to ensure that they are fully familiar with any device they choose to implant to minimise this risk.

### Bleeding Complications

Bleeding may occur secondary to anticoagulation given during or following the stenting procedure, or from vessel perforation or rupture. These can range from minor (epistaxis, haematuria) to major (cerebral bleeding leading to stroke). Small vessel bleeds may go unnoticed during the procedure and result in a retroperitoneal or groin haematoma. Iliac, IVC or spinal vessel rupture are rare but can be catastrophic and result in paraplegia [31, 32] or death. Small, access site bleeds in patients who are not on systemic thrombolysis can be managed conservatively, and patients do not usually need to stop anticoagulation. Referral to haematology is essential for patients with more significant bleeding, and the risks of stent thrombosis/occlusion from stopping anticoagulation must be balanced with the risks of an ongoing bleed, which may require transfusion, reversal of anticoagulation or surgical evacuation of the haematoma.

Bleeding complications are most common in patients who are undergoing catheter-directed thrombolysis (CDT), and can occur at any site, including the venous puncture sites, bowel, bladder, intracranial, or any recent surgical intervention site (for example epidural puncture for analgesic purposes). The risk of bleeding secondary to CDT can be reduced with robust protocols for patient monitoring to enable early recognition of bleeding and guide temporary cessation of therapy. CDT can be avoided altogether for patients being treated for acute DVT with the use of mechanical thrombectomy devices. However, some of these are contraindicated in patients who already have a stent in place.

Iliac vein or IVC rupture during endovascular treatment for venous outflow obstruction is extremely rare. This complication has been described in a small number of case studies [33–35] and can be managed with the placement of a covered or bare-metal stent, and resuscitation of the patient with fluids and blood products as required. In patients with multiple large spinal collaterals, care must be taken to ensure that the wire does not traverse these vessels when there is difficulty in passing through the iliofemoral segment. Position must be checked before balloon angioplasty begins, and multiple views with lateral and oblique angles can be helpful to confirm that the wire is correctly placed. In the case of inadvertent rupture of the spinal vessels, neurological complications can occur, and these patients require urgent neurosurgical referral.

### Thromboembolic Complications

Pulmonary embolism during or in the postoperative period is rare if patients are adequately anticoagulated. A small number of cases have been reported in the literature [5, 6, 36–38], with most occurring in patients with underlying malignancy or other pro-thrombotic risk factors. Anticoagulation must be carefully managed in such patients. Contralateral iliofemoral DVT has been reported in older case series using the Wallstent, this is likely a result of the recommended deployment technique of the stent, which requires jailing against the contralateral wall of the IVC. This is necessary because of the design of the stent, which is weaker at the edges than the main body and therefore prone to compression or collapse if landed in venous segments that are diseased or externally compressed. This jailing in the IVC can lead to flow disturbance in the contralateral iliac vein [39]. Dedicated venous stents have been designed with uniform radial resistive force and crush resistance to avoid this issue with deployment, and whiile the available follow-up data for these devices is of shorter duration than the Wallstent, initial experience suggests that contralateral DVT is less common. When bilateral stenting is required, extension into the IVC with double-barrelled stenting [40] or Z-stenting can be used to avoid jailing.

In-stent thrombosis may occur in the early (24 h–2 weeks) postoperative period. In some cases, the beginnings of thrombus formation inside the stent can be seen with IVUS immediately after the stent is placed. Early stent surveillance with duplex ultrasound can help to identify patients with stents that are at risk for early thrombosis, and at our centre all patients will have a check DUS prior to discharge, and at two weeks postprocedure. A reduction in stent diameter of > 75% at 24 h suggests impending stent occlusion and at our centre is an indication for urgent re-intervention, which is usually with catheter-directed thrombolysis and adjuvant pharmacomechanical or mechanical thrombectomy, with devices such as the Angiojet Zelante™ (Boston Scientific) or Inari ClotTriever®. If repeated in-stent thrombosis occurs, the patient’s anticoagulation should be discussed with the haematology team, anti-Xa levels checked for patients who have been initially anticoagulated with low molecular weight heparin and unusual situations such as heparin induced thrombocytopenia should be considered. The role of antiplatelets in venous stent thrombosis has not been fully established but may be of benefit in patients with repeated occlusion [41]. Risk factors for early stent occlusion can be categorised as related to disease of the inflow vessels, haematological issues (poor anticoagulation control) or technical errors such as missed inflow or outflow disease [42]. These should be optimised before stenting through multi-disciplinary discussions with haematology for anticoagulation management, monitoring of intraoperative anticoagulation through ACT (activated clotting time), consideration of adjuvant procedures to maintain flow through the stent system for patients with severe inflow disease, for instance, endoplebectomy and AV fistula. Correct stent sizing and thorough evaluation of the lesion before intervention to determine the length of coverage required to stent from healthy vessel to healthy vessel are also essential to reduce the risk of early stent thrombosis.

### Back Pain

Low-back pain is frequently encountered after stenting, the aetiology of this is poorly understood but may be related to compression of the lower lumbar and upper sacral nerve roots or stretching of the vein wall [43], Back pain is expected in the immediate postoperative period and should improve in the first 2–4 weeks after the intervention, although this can become chronic in some cases [43, 44]. Patients should be appropriately counselled regarding this pre-opratively. Dilatation of chronic iliocaval venous obstruction can be extremely painful for patients, and for this reason, procedures are carried out under general anaesthetic at our centre. Patients may still experience severe back pain in the immediate postoperative period even with general anaesthesia, which can be managed with opioid analgesia, and in our centre ketamine has been used in recovery to good effect for severe cases. If patients continue to have severe pain, patient-controlled analgesia (PCA) may be necessary for the first 24–48 h postoperatively. Oversizing of stents may increase the risk of chronic back pain so it is important to select stents of appropriate diameter, which in most cases will be < 16 mm. Referral to pain management specialists is the mainstay of treatment in cases of chronic pain, with various pharmacological and mechanical (e.g. neuromodulation) therapies available.

### Compression of Adjacent Vessels

**Fig. 3:**
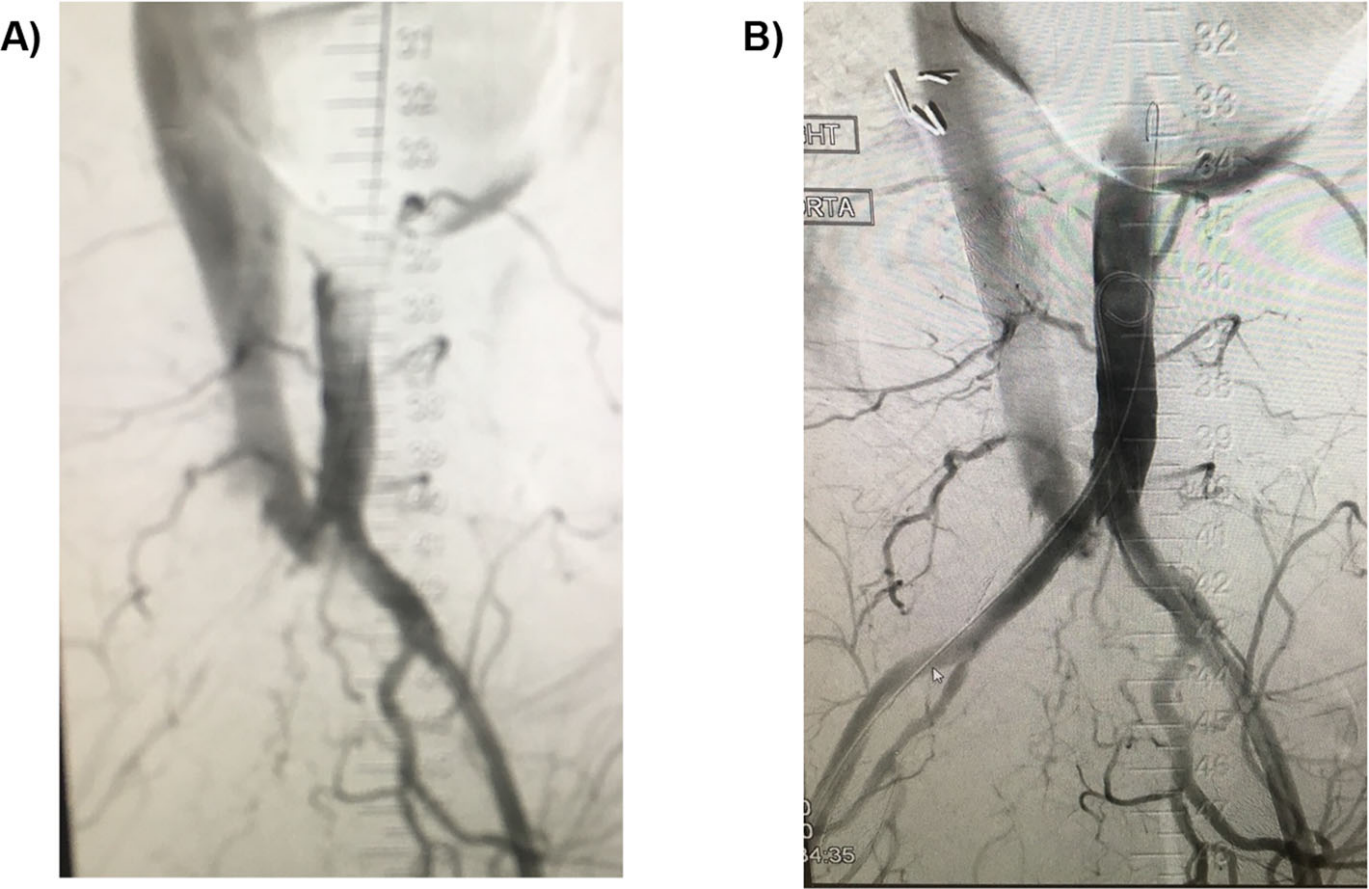
Occluded right common iliac artery after compression from an IVC stent in a retroperitoneal fibrosis patient with an associated arterial-caval fistula. **A** Pre- and **B** post-treatment of the right common iliac artery with a covered stent. Fistula was absent on CT scan the following day

In venous interventions, stents typically expand without causing issues to adjacent structures however when dealing with reconstruction in patients, where the underlying disease process is associated with retroperitoneal abnormalities this may occur. In patients with retroperitoneal fibrosis compression of the adjacent arterial vessels (iliac artery and right renal) may occur [1] (Fig. 3), which may be treated with an additional stent.

## Summary

Complications of venous stenting are rare, and mostly avoidable. Of the potential complications, stent migration is arguably the most feared. This cannot happen if a stent is not placed. Patient selection is therefore crucial, and thorough evaluation of the iliofemoral venous system with multiple imaging modalities will determine which patients will benefit from stenting. When stenting is required, correct sizing and the identification of an appropriate landing zone are essential both to prevent migration and to maintain stent patency. Close follow-up of patients, particularly in the early postoperative period is important to identify those who may be developing in-stent-thrombosis. Device-related complications such as fractures may reduce over time as stent designs continue to improve, however appropriate placement is again necessary to minimise the risk of these occurring.
